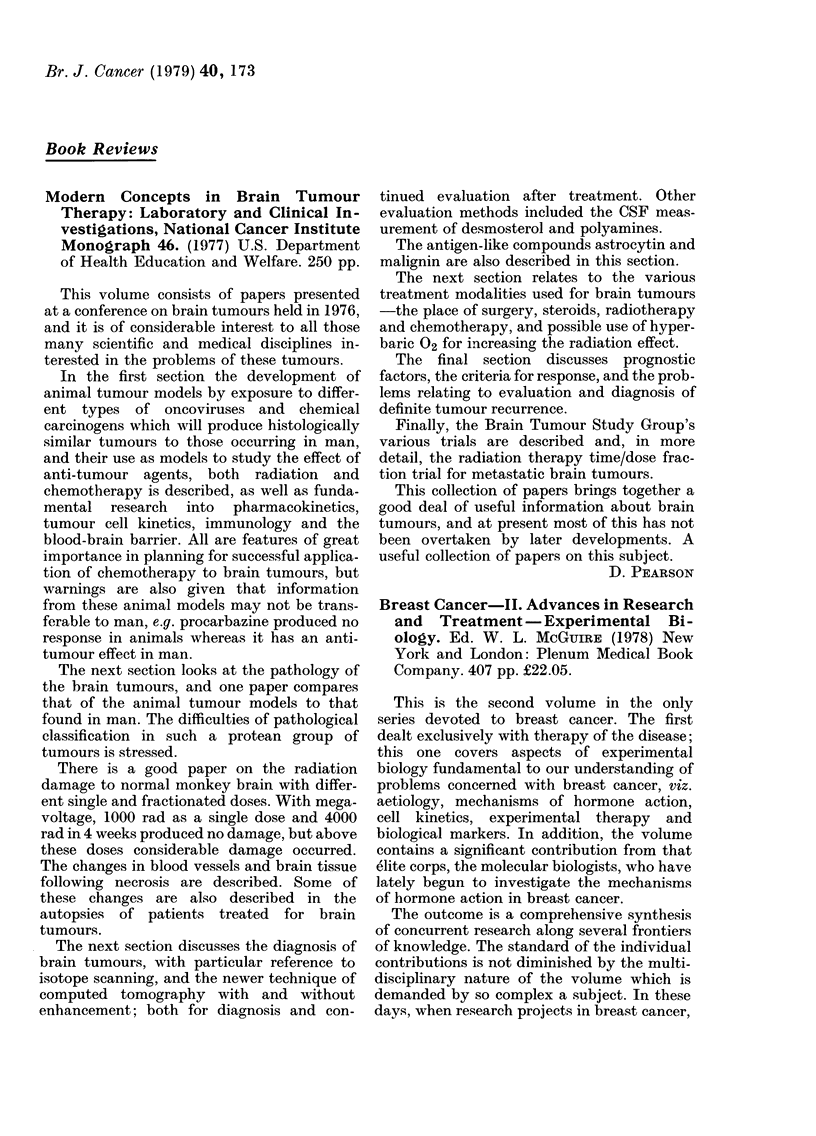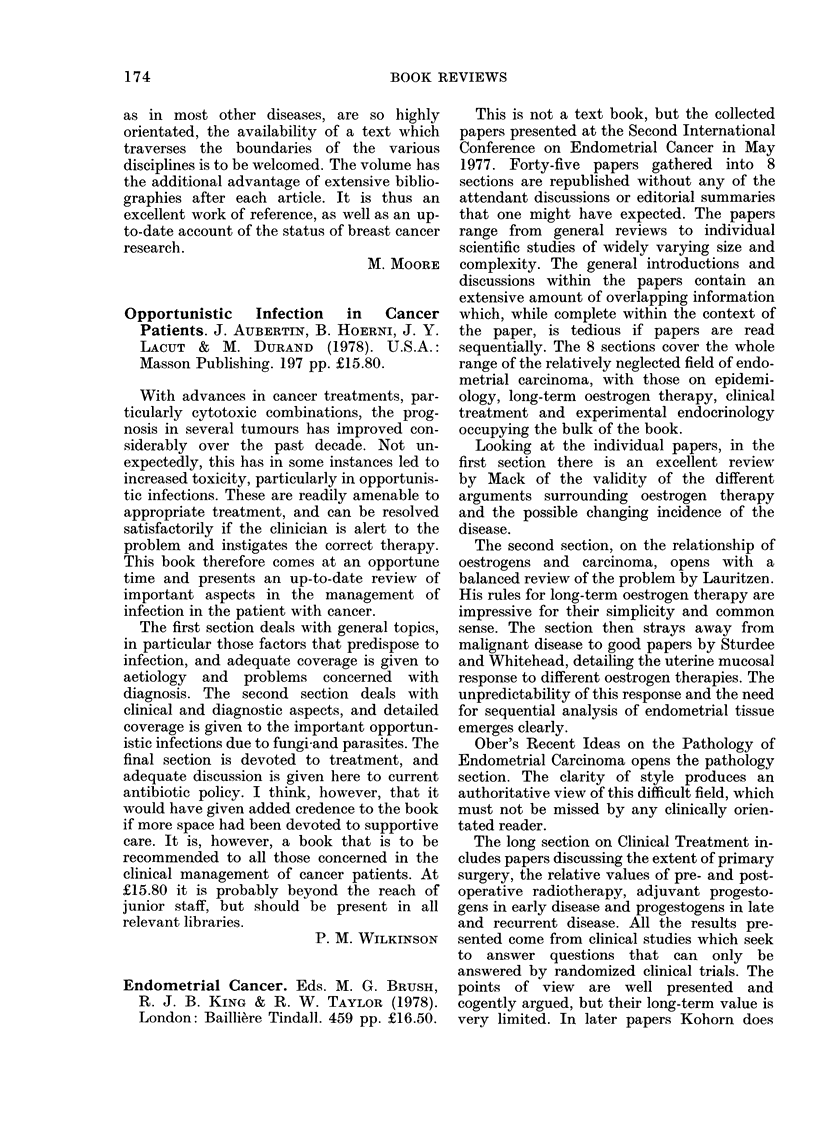# Breast Cancer—II. Advances in Research and Treatment—Experimental Biology

**Published:** 1979-07

**Authors:** M. Moore


**Breast Cancer—II. Advances in Research and Treatment—Experimental Biology.** Ed. W. L. McGUIRE (1978) New York and London: Plenum Medical Book Company. 407 pp. £22.05.

This is the second volume in the only series devoted to breast cancer. The first dealt exclusively with therapy of the disease; this one covers aspects of experimental biology fundamental to our understanding of problems concerned with breast cancer, *viz.* aetiology, mechanisms of hormone action, cell kinetics, experimental therapy and biological markers. In addition, the volume contains a significant contribution from that élite corps, the molecular biologists, who have lately begun to investigate the mechanisms of hormone action in breast cancer.

The outcome is a comprehensive synthesis of concurrent research along several frontiers of knowledge. The standard of the individual contributions is not diminished by the multidisciplinary nature of the volume which is demanded by so complex a subject. In these days, when research projects in breast cancer, as in most other diseases, are so highly orientated, the availability of a text which traverses the boundaries of the various disciplines is to be welcomed. The volume has the additional advantage of extensive bibliographies after each article. It is thus an excellent work of reference, as well as an up-to-date account of the status of breast cancer research.

M. Moore